# Long noncoding RNA as a biomarker for the prognosis of ischemic stroke: A protocol for meta-analysis and bioinformatics analysis

**DOI:** 10.1097/MD.0000000000025596

**Published:** 2021-04-30

**Authors:** Jing Fu, Qian Yu, Jun Xiao, Suping Li

**Affiliations:** a Department of Rehabilitation; b Department of Neurology, Sichuan Academy of Medical Sciences & Sichuan Provincial People's Hospital, Chengdu, Sichuan Province, China.

**Keywords:** bioinformatics, ischemic stroke, long noncoding RNA, meta-analysis, prognosis, protocol

## Abstract

**Background:**

As the most common type of cerebrovascular disease, ischemic stroke is the disturbance of cerebrovascular circulation caused by various factors, with complex pathogenesis. At present, the molecular mechanism of ischemic stroke is still unclear, and there lacks early diagnostic markers. Therefore, there is an urgent need to find effective preventive measures, active diagnostic methods and rapid treatment measures. In recent years, related studies have displayed that long noncoding RNAs (lncRNAs) is related to the prognosis of ischemic stroke. However, the results are not supported by some evidence. Therefore, in this study, meta-analysis was used to analyze the relationship between lncRNAs and the prognosis of ischemic stroke. In addition, we carried out bioinformatics analysis to study the action mechanism and related pathways of lncRNAs in ischemic stroke.

**Methods:**

Literature search was operated on databases up to March 2021, including China National Knowledge Infrastructure, Chinese Biomedical literature Database, Chinese Scientific and Journal Database, Wan Fang database, Web of Science, PubMed, and EMBASE. The relationship between lncRNAs expression and survival outcome was estimated by hazard ratio (HR) and 95% confidence interval (CI). Meta-analysis was conducted on the Stata 16.0. Starbase v2.0 software predicts microRNAs (miRNAs) that interacts with lncRNAs. In addition, HMDD v2.0 database filters out miRNAs related to ischemic stroke. Furthermore, Consite transcription factor database was used to predict the transcription factors of each lncRNAs and miRNA. At the same time, the transcription factors related to ischemic stroke were screened out after intersection. miRwalk online software was applied to predict the target mRNA of each miRNA, and the common target genes were screened by consistent method. The molecular regulatory network map of lncRNAs in ischemic stroke was drawn. Based on the overlapping target genes, gene ontology (GO), Kyoto Encyclopedia of Genes and Genomes (KEGG), and protein-protein interaction (PPI) network analysis were carried out to explore the possible mechanism.

**Results:**

The results of this meta-analysis would be submitted to peer-reviewed journals for publication.

**Conclusion:**

This study will provide evidence-based medical evidence for the relationship between lncRNA and the prognosis of ischemic stroke. What is more, bioinformatics analysis will provide ideas for the study of ischemic stroke mechanism.

**Ethics and dissemination:**

The private information from individuals will not be published. This systematic review also should not damage participants’ rights. Ethical approval is not available. The results may be published in a peer-reviewed journal or disseminated in relevant conferences.

**OSF Registration Number:**

DOI 10.17605/OSF.IO/QBZW6.

## 1 Introduction

Ischemic stroke is a kind of acute cerebrovascular disease with high morbidity and mortality, causing about 700,000 deaths or disabilities worldwide every year.^[1]^ It affects the life quality of about 795,000 people every year and places a serious burden on patients, their families, and even the society.^[2]^ The pathogenesis of ischemic stroke is complex, including the results of the interaction between genes and environmental factors. At present, thrombolysis is an effective method for the treatment of ischemic stroke. But it will inevitably lead to reperfusion injury of nerve cells. Therefore, the targeted regulation of biomarkers of ischemic stroke is very important for the treatment of ischemic stroke.

Long non-coding RNAs (lncRNAs) is a kind of non-coding RNA with a length of more than 200 bp.^[3]^ At present, more than 15,000 kinds of lncRNAs have been found in humans, but few is known about their structure and function.^[4]^ LncRNA participates in biological processes such as proliferation, differentiation, apoptosis and autophagy, and regulates expression at transcriptional and post-transcriptional levels. Current studies have revealed that lncRNA is related to the occurrence and development of many diseases, such as tumor, cardiovascular disease, endocrine disease, and so on.^[5–7]^ Additionally, lncRNA is also a good biomarker of tumor prognosis.^[8,9]^

In recent years, hundreds of abnormally expressed lncRNA have been discovered in ischemic stroke patients and animal models.^[10,11]^ LncRNA is closely related to ischemic stroke and has the potential to become a biomarker and therapeutic target of ischemic stroke. In recent years, studies have shown that the expression level of lncRNA can be regarded as a biomarker of prognosis and diagnosis.^[12–16]^ In order to more accurately analyze the effects of LncRNA on the prognosis of patients with ischemic stroke, a meta-analysis was conducted to evaluate the relationship between lncRNA and the prognosis of ischemic stroke. Most importantly, this study used bioinformatics methods to explore the regulatory relationship between lncRNA and upstream transcription factors, and downstream target microRNA (miRNA) and miRNA target genes. At the same time, bioinformatics analysis was carried out, including gene ontology (GO), Kyoto Encyclopedia of Genes and Genomes (KEGG), and the protein-protein interaction (PPI) network analysis. Through the above analysis methods, we can better understand the mechanism of lncRNA in terms of the occurrence and development of ischemic stroke, thus providing clues for further experiments to verify its molecular regulatory network mechanism.

## 2 Methods

### 2.1 Study registration

This meta-analysis protocol is based on the Preferred Reporting Items for Systematic Reviews and meta-analysis Protocols (PRISMA-P) statement guidelines.^[17]^ The protocol of the systematic review was registered on Open Science Framework, and the registration number is DOI 10.17605/OSF.IO/QBZW6.

### 2.2 Data sources and retrieval strategy

We searched the China National Knowledge Infrastructure, Chinese Biomedical literature Database, Chinese Scientific and Journal Database, Wan Fang database, Web of Science, PubMed, and EMBASE databases to identify all potentially eligible articles from inception to March 2021. The detailed search strategies are listed in Table 1. Furthermore, the reference lists from retrieved studies were conducted to identify all potentially eligible articles.

**Table 1:** Search strategy in PubMed database.

Number	Search terms
#1	RNA, Long Untranslated[MeSH]
#2	LINC RNA[Title/Abstract]
#3	LincRNAs[Title/Abstract]
#4	Long intergenic non-protein coding RNA[Title/Abstract]
#5	Long non-coding RNA[Title/Abstract]
#6	Long non-protein-coding RNA[Title/Abstract]
#7	Long noncoding RNA[Title/Abstract]
#8	Long ncRNA[Title/Abstract]
#9	Long ncRNAs[Title/Abstract]
#10	RNA, long non-translated[Title/Abstract]
#11	Long intergenic non protein coding RNA[Title/Abstract]
#12	Long non coding RNA[Title/Abstract]
#13	Long non protein coding RNA[Title/Abstract]
#14	Long non-translated RNA[Title/Abstract]
#15	Long untranslated RNA[Title/Abstract]
#16	Non-coding RNA, long[Title/Abstract]
#17	Non-protein-coding RNA, long[Title/Abstract]
#18	Non-translated RNA, long[Title/Abstract]
#19	Noncoding RNA, long[Title/Abstract]
#20	RNA, long non translated[Title/Abstract]
#21	RNA, long non-coding[Title/Abstract]
#22	RNA, long non-protein-coding[Title/Abstract]
#23	RNA, long noncoding[Title/Abstract]
#24	Untranslated RNA, long[Title/Abstract]
#25	ncRNA, long[Title/Abstract]
#26	ncRNAs, long[Title/Abstract]
#27	or/1-26
#28	Stroke[MeSH]
#29	Apoplexy[Title/Abstract]
#30	Cerebral stroke[Title/Abstract]
#31	Cerebrovascular accident[Title/Abstract]
#32	Cerebrovascular apoplexy[Title/Abstract]
#33	Vascular accident, brain[Title/Abstract]
#34	CVA (Cerebrovascular accident)[Title/Abstract]
#35	Cerebrovascular accident, acute[Title/Abstract]
#36	Cerebrovascular stroke[Title/Abstract]
#37	Stroke, acute[Title/Abstract]
#38	Acute cerebrovascular accident[Title/Abstract]
#39	Acute cerebrovascular accidents[Title/Abstract]
#40	Acute stroke[Title/Abstract]
#41	Acute strokes[Title/Abstract]
#42	Apoplexy, cerebrovascular[Title/Abstract]
#43	Brain vascular accident[Title/Abstract]
#44	Brain vascular accidents[Title/Abstract]
#45	CVAs (Cerebrovascular accident)[Title/Abstract]
#46	Cerebral strokes[Title/Abstract]
#47	Cerebrovascular accidents[Title/Abstract]
#48	Cerebrovascular accidents, Acute[Title/Abstract]
#49	Cerebrovascular strokes[Title/Abstract]
#50	Stroke, cerebral[Title/Abstract]
#51	Stroke, cerebrovascular[Title/Abstract]
#52	Strokes[Title/Abstract]
#53	Strokes, acute[Title/Abstract]
#54	Strokes, cerebral[Title/Abstract]
#55	Strokes, cerebrovascular[Title/Abstract]
#56	Vascular accidents, brain[Title/Abstract]
#57	or/28-56
#58	Prognos∗[Title/Abstract]
#59	Survival [Title/Abstract]
#60	or/58–59
#61	#27 and #57 and #60

### 2.3 Inclusion criteria for study selection

#### 2.3.1 Inclusion criteria

1. The patients who were diagnosed with ischemic stroke;
2. The samples of lncRNA come from platelets, serum, peripheral blood mononuclear cells, plasma, and whole blood;
3. The relationship between the expression of lncRNA and the prognosis of patients with ischemic stroke was analyzed. Prognostic indicators include overall survival (OS) or recurrence-free survival;
4. Sufficient data were included to extract or calculate the hazard ratio (HR).

#### 2.3.2 Exclusion criteria

1. Repeatedly published research;
2. Animal experiment;
3. Comments, case reports, conference summaries and meta-analysis;
4. Insufficient data were extracted or calculated for HR and its 95% confidence interval (CI).

### 2.4 Data collection and analysis

**Figure 1:**
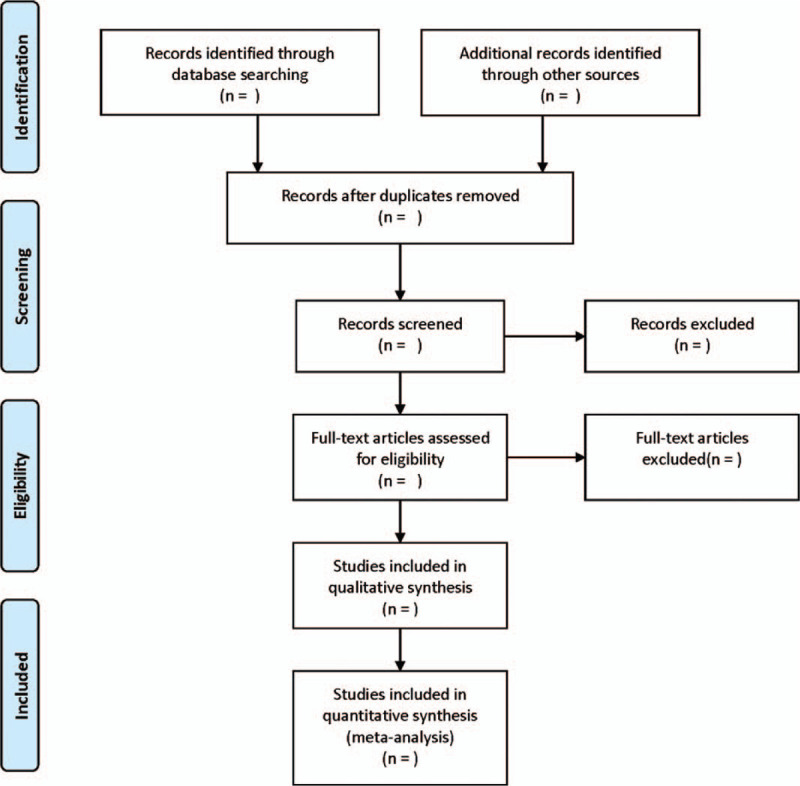
Flow diagram of study selection process.

The literature screening process is displayed in Figure 1. First of all, the abstracts and topics were read independently by 2 researchers. According to the inclusion criteria and exclusion criteria, preliminary screening was carried out. Finally, the included literature was sorted out and the data are extracted from the included research. The data collected include the first author, the number of years published, the nationality of the study, the design of the study, the source of the sample, the detection method, the type of lncRNA, the longest follow-up period, outcome indicators, multivariate analysis, etc. Furthermore, in view of the fact that some studies only provide Kaplan–Meier curves, it is necessary to use Engauge Digitizer4.1 version to extract HR and its 95% CI from graphic survival curves.^[18,19]^ If there exist different opinions, it will be determined through discussion by a third researcher.

### 2.5 Quality assessment

The quality of the included references was assessed using the Newcastle–Ottawa Scale (NOS).^[20]^ The maximum range of quality score is 10. The more stars of the score are, the better the quality of the research literature is. Score ≥6 indicates that the quality of the literature is high.^[21]^ The above processes were completed independently by 2 researchers. If there are different opinions, it will be settled through negotiation or by a third researcher.

### 2.6 Measures of prognosis

Overall survival or recurrence-free survival was taken as prognostic outcomes, and the results were expressed as HRs with 95% CIs.

### 2.7 Management of missing data

If there exists insufficient or missing data in the literature, we would only analyze the currently available data and discuss its potential value.

### 2.8 Statistical analysis

Meta-analysis was conducted on the Stata 16.0 (Stata Corporation, TX). HR and its 95% CIs were used to evaluate the relationship between lncRNA expression and prognosis in patients with ischemic stroke. Heterogeneity was tested by Q-statistic and *I*^*2*^-statistic, *I*^*2*^ > 50% was considered as significant heterogeneity, and the random-effects model or the fixed-effects model was used. *P* values in this study were two-sided, and *P* < .05 indicated that there was a statistical significance.

### 2.9 Additional analysis

#### 2.9.1 Subgroup analysis

According to the detection methods of LncRNA, ethnicity, and the source of survival data, we analyzed the subgroup.

#### 2.9.2 Sensitivity analysis

If the overall pooled estimation was stable, sensitivity analysis was performed via sequential deletion of a single included study to test.

#### 2.9.3 Reporting bias

Egger linear regression test and Begg rank test were used to assess publication bias.^[22,23]^

## 3 Bioinformatics analysis

### 3.1 Predicting miRNAs related to lncRNAs

Based on the lncRNAs obtained from the meta-analysis, we do the following analysis. The miRNAs related to lncRNAs can be predicted by using Starbasev2.0 online software (http://starbase.sysu.edu.cn/starbase2/index.php). Entering the “miRNA-lncRNA” regulatory network prediction interface through “miRNA Target,” and the miRNAs with the combination of prediction can be obtained after submitting the target lncRNA.

### 3.2 Querying confirmed miRNAs related to ischemic stroke

HMDD v3.2 (http://www.cuilab.cn/hmdd/) query for proven ischemic stroke-related miRNAs.

### 3.3 Filtering miRNA related to lncRNAs and ischemic stroke

miRNAs related to lncRNAs and ischemic stroke were intersected and the common miRNAs were screened out.

### 3.4 Prediction of lncRNAs-related transcription factors

The transcription factors of each lncRNA were predicted in Consite database (http://consite.genereg.net/) and their common transcription factors were screened by intersection. The regulatory mechanism of transcription factors in ischemic stroke was investigated through PubMed.

### 3.5 Prediction of transcription factors for each miRNA

The transcription factors of each miRNA were predicted and analyzed by Consite database (http://consite.genereg.net/). The common transcription factors of the above-mentioned miRNAs were screened. The relationship between transcription factors and ischemic stroke was queried by PubMed database.

### 3.6 Predicting the target mRNA of each miRNA

The target mRNA of the above miRNAs was queried by miRwalk v2.0 (http://zmf.umm.uni-heidelberg.de/apps/zmf/mirwalk2/) and the common target genes were screened after intersection. The common target genes related to ischemic stroke were screened by PubMed database.

### 3.7 Drawing the network regulation diagram

Based on the above results, the regulation network diagram of lncRNA in ischemic stroke is drawn by using Cytoscape software.

### 3.8 GO and KEGG analysis

According to the common target genes selected above, we analyzed the GO function and KEGG pathway through the online tool DAVID database (https://david.ncifcrf.gov/).

### 3.9 Protein-protein interaction

The screened common target genes were constructed by String website (https://www.string-db.org/) to construct PPI network analysis.

## 4 Ethics

Our research data were derived from published literatures, because there were no patient recruitment and personal information collection. Therefore, ethical approval was not required.

## 5 Discussion

Ischemic stroke is mainly caused by cerebral pharmacies, which are mainly blood vessels and neuroprotection.^[24]^ At present, the only clinically effective revascularization drug approved by FDA is recombinant tissue plasminogen activator (rtPA). Due to its narrow treatment time window, its application is greatly limited.^[25,26]^ At present, it has not been proved that neuroprotective drugs have definite clinical effectiveness and is not enough to treat ischemic stroke, so it is of great significance to find new therapeutic targets.^[27]^

LncRNA is involved in the pathological process of ischemic stroke by a variety of mechanisms, including inflammation, apoptosis and autophagy, arteriosclerosis, neurogenesis, and so on,^[28–30]^ which suggests that LncRNA has high biological value in the diagnosis and prognosis of ischemic stroke and is expected to become a new therapeutic target. In this study, meta-analysis and a variety of bioinformatics analysis methods were adopted to further explore the biological role and molecular mechanism of lncRNA, so as to provide a theoretical basis for the prognosis of ischemic stroke.

## Author contributions

**Conceptualization:** Suping Li.

**Data curation:** Jing Fu, Qian Yu.

**Formal analysis:** Jing Fu.

**Funding acquisition:** Jing Fu, Suping Li.

**Methodology:** Qian Yu.

**Project administration:** Jing Fu, Suping Li.

**Resources:** Jing Fu.

**Software:** Qian Yu.

**Supervision:** Qian Yu.

**Validation:** Qian Yu, Jun Xiao.

**Visualization:** Jun Xiao.

**Writing – original draft:** Suping Li, Jing Fu.

**Writing – review & editing:** Suping Li, Jing Fu.
